# Knowledge, attitude and practices related to COVID-19 among young Lebanese population

**DOI:** 10.1186/s12889-021-10575-5

**Published:** 2021-04-06

**Authors:** Samer Sakr, Ali Ghaddar, Imtithal Sheet, Ali H. Eid, Bassam Hamam

**Affiliations:** 1 Department of Biological and Chemical Sciences, School of Arts and Sciences, Lebanese International University, Beirut, Lebanon; 2 Department of Biomedical Sciences, School of Arts and Sciences, Lebanese International University, Beirut, Lebanon; 3 Department of Basic Medical Sciences, College of Medicine, QU Health, Qatar University, Doha, Qatar; 4 Biomedical and Pharmaceutical Research Unit, QU Health, Qatar University, Doha, Qatar

**Keywords:** COVID-19, KAP, Knowledge, Attitude, Practices and Lebanon

## Abstract

**Background:**

As the world faces the most serious and widespread pandemic in recent history, claiming nearly 1,945,610 lives and infecting over 90 million individuals up to January 13, 2021, controlling the spread of COVID-19 is still limited to efforts done by the general population implementing rules and restrictions passed by world governments and organizations. As we wait for the approved vaccines to become widely distributed, the best approach to fighting the spread of this disease is mostly preventative depending largely on individuals’ compliance. This study aimed to determine the knowledge, attitude and practices (KAP) towards COVID-19 in Lebanon.

**Methods:**

A descriptive analysis was performed to describe the outcome measures of knowledge, attitudes and practices towards COVID-19 on a convenience sample from the Lebanese population in relation to socio-demographic characteristics and level of concern towards COVID-19. One thousand eight hundred sixty-one participants filled in an online survey (response rate: 18.5%) distributed by social media to social networks of the research team members.

**Results:**

Participants were mainly young (49.4% between 18 and 24 years) and males (73.7%). Participants showed an overall appropriate knowledge of COVID-19 (67.1%) and positive attitude (around 90% were optimistic about treatment and vaccination) and had good preventive practices towards COVID-19 (around 75% washed hands and avoided public places). Knowledge and practices correlated positively with marriage, age, education, working in a healthcare field and with the level of concern about getting COVID-19.

**Conclusions:**

This study found good overall levels of KAP among the studied Lebanese population. This can help in controlling the spread of COVID-19 if individuals were forced to adhere to social distancing and appropriate preventative practices.

**Supplementary Information:**

The online version contains supplementary material available at 10.1186/s12889-021-10575-5.

## Background

The world is currently facing a serious pandemic caused by a novel coronavirus that was first isolated on December 21, 2019 [1]. The disease was spread from patients who were in contact with the Huanan Seafood Market (in Wuhan, China), which is widely known to be the origin of this pandemic [2]. This virus has since been named severe acute respiratory syndrome coronavirus 2 (SARS-CoV-2) following the most recent World Health Organization (WHO) guidelines [3].

Coronavirus disease 2019 (COVID-19), which is caused by SARS-CoV-2, is reported by the WHO to be triggered by the inhalation of contaminated droplets with viral particles or by touching the nose, mouth and eyes after a person’s hands come in contact with contaminated surfaces [4, 5]. In addition, transmission via aerosols and through fecal-oral routes are also suspected. Among the most described symptoms of COVID-19 are fever, cough, sore throat, shortness of breath, pneumonia, fatigue, malaise and gastrointestinal symptoms. At the same time, infection by the SARS-CoV-2 can be asymptomatic [6].

Up to January 13, 2021, a worldwide total of 90,054,813 confirmed infected cases with SARS- CoV-2 were reported by the WHO, out of which, 1,945,610 deaths were confirmed, constituting a mortality rate of 2.16% [7]. In the eastern Mediterranean region, WHO reported 5,222,466 cases and 126,042 deaths (mortality rate of 2.41%). The first confirmed case infected with SARS-CoV-2 was diagnosed in Lebanon on February 21, 2020 where, up to January 13, the total number of people positive for SARS-CoV-2 was 226,948 with 1705 total deaths (0.75% mortality rate) [7]. In Lebanon, during the months of November and December 2020, on average, 1614 new confirmed cases per day was reported by the WHO. From January 1 to January 13, 2021, these numbers have alarmingly risen to 3763 new cases per day signaling an increasingly alarming situation in this country [7].

Shortly after the pandemic reached Lebanon, the Lebanese Ministry of Public Health (MOPH) devised a policy to control the spread of the SARS-CoV-2 within the population which was in alignment with those of the WHO [8, 9]. The published policy regarding the general population are summarized hereafter: i) regularly and thoroughly clean your hands with an alcohol-based hand rub or wash them with soap and water, ii) maintain at least 1 m (3 ft) distance between yourself and others, iii) avoid going to crowded places, iv) avoid touching eyes, nose and mouth, v) cover mouth and nose with a bent elbow or tissue when you cough or sneeze, then dispose of the used tissue immediately and wash your hands, vi) stay home and self-isolate even with minor symptoms such as cough, headache and mild fever, until you recover while asking someone to bring you the needed supplies, vii) wear a mask to avoid infecting others if you need to leave your house, viii) seek medical attention, call by telephone in advance if possible and follow the directions of your local health authority if you have a fever, cough and difficulty breathing [8, 9].

Although a number of vaccines have been developed, their delivery and administration to the entire world population is still far from being achieved. At the same time, many of the available tests to detect for SARS- CoV-2 are still not very accurate, with yet high percentages of false negatives/positives results depending on the test types [10, 11]. With this in mind, prevention seems to be the only actionable plan at the moment to slow down the spread of this virus. Prevention naturally depends on controlling the spread of the infection from infected patients to the general population. This requires clear and decisive measures by governments and communities on (1) how to correctly identify COVID-19 patients, (2) how to quarantine and treat them and (3) how to educate the populace about appropriate measures to control its spread. This requires appropriate KAP at the level of healthcare workers (HCW) as well as the general populace [12, 13]. Many studies have shown that healthcare workers show adequate KAP towards viruses of the respiratory system including COVID-19 [12–14]. This is not surprising since HCW have better access to scientific information and better KAP related to the spread of infectious diseases. At the same time, HCW have better access to protective gear as provided by their workplace. Therefore, this study aims to assess the KAP among the young Lebanese population in order to gain a better understanding of the current KAP indicators and the gaps thereof. A study that would aid in the design of public policies to control COVID-19 spread until a cure or vaccine is readily available to the general public.

## Methods

### Study design and data collection

This descriptive study was based on a cross-sectional survey conducted between April 10 and May 5, 2020 in Lebanon. A structured questionnaire was designed and developed by the research team. Some questions were based on previous studies targeting other disease, such as malaria and hepatitis B, with the needed adjustment to fit the characteristics of COVID-19 [15, 16], and others were self-created by the research team.

A pilot study on 22 individuals was conducted to check the reliability of the questionnaire. Based on the results of the pilot study, modifications have been applied to adjust the questionnaire based on the Lebanese context. The questionnaire reliability was assessed by calculating the Cronbach’s alpha’s coefficients, which were acceptable for the three dimensions of the questionnaire (knowledge: Cronbach’s alpha = 0.61, attitude: Cronbach’s alpha = 0.66 and practices: Cronbach’s alpha = 0.64).

An estimated sample size of 1153 participants were calculated based on a 95% confidence level with population = 6 million, an alpha-error of ±2.5% and an estimated prevalence of knowledge about COVID-19 of 75% based on previous studies in the region [17, 18]. The questionnaire was shared through social media platforms (Facebook, Instagram and LinkedIn) through the accounts of the members of the research team with all our contacts and friends. In addition, members of the research team shared the survey link through our E-mail lists and contacts in the chatting group “WhatsApp”. We were able to keep track of the number of responses to our survey through the survey’s google form “responses” section. We also sent one or two reminders to our contacts to encourage them to fill out our survey. We estimated 10,000 individuals to whom the survey link was sent, who comprised the study sample. In many cases, some of our friends shared the invite to this study on their accounts as well. This fact made it difficult to collect the total number of individuals who could have received this questionnaire in order for us to calculate the response rate for our study. A total of 1861 individuals responded to the questionnaire (response rate around 19%). Missing data was handled using complete case (aka listwise deletion).

As there were no previous studies in Lebanon about KAP towards COVID-19, and in light of the rapid evolving context of the pandemic and the need to develop rapid measures to contain the spread of the virus, we chose to select a convenience sample of young participants from all regions in Lebanon. In addition, as the research team had the priority to avoid any risk of infection during data collection, we chose the data collection by social media to avoid any direct contact in this phase.

### Variables

The questionnaire consisted of four sections: socio-demographics, KAP towards COVID-19. Participants’ socio-demographic characteristics consisted of 6 questions including gender (female, male), age (18–24, 25–34, 35–44, 45–54, 55–64, 65 or more), marital status (single, married, divorced, widowed) residence (rural, urban), education (elementary or less, high school, university, postgraduate) and employment (unemployed, student, health field, office work, construction).

Knowledge consisted of 10 questions including the source of SARS-CoV-2 virus (human, animal, engineered in the laboratory, etc.…), transmission routes (handshakes and kisses, face to face talk, blood transfusion, etc.…), sources of information (WHO, TV, social media, etc.…), targeted human system (respiratory, digestive, blood, etc.…), incubation period (14 days, 6–7 days, etc.…), possible symptoms (vomiting, fever, headache, fatigue, etc.…), patient’s recovery, risk groups (children, people above 60 years, etc.…) and 2 questions related to assessing the relation between symptoms and vaccine of corona virus with influenza viruses. An example of questions about knowledge was “How is COVID-19 transmitted from one person to another?”. Attitude section of the questionnaire consisted of 7 questions for example “Are you worried that you or one of your family members would catch the COVID- 19?”, and practices section was composed of 10 questions such as “Are you disinfecting food you are buying during the COVID-19 pandemic?”. The complete questionnaire is found as supplementary material 1.

For the questions related to knowledge, participants scored 0 on each question with the wrong answer and 1 for each question with the right answer in all questions except the 3 questions that included more than one correct answer. In these 3 questions, participants scored 1 (good knowledge) if they answered 3 or more out of 5 correct answers, scored 0.5 (average knowledge) if they answered 1–2 correct answers and scored 0 (poor knowledge) if they did not guess any of the correct answers. Answers to questions related to frequency of practices were scored on a 4-point Likert scale (1: always and 4: never). The sum of scores was calculated for the two dimensions of knowledge and practices considering the sum of the score for each individual question in each dimension.

### Statistical analysis

Descriptive statistics showed the frequency and percentage (%) of participants who answered correctly on the different questions related to KAP towards antibiotics use. The non-parametric Mann-Whitney U test and the Kruskal-Wallis test were used, since normality could not be assumed, to compare the average score in the two dimensions of knowledge and practices between the participants according to their socio-demographic characteristics and according to their attitudes (worried or not about getting infected with COVID-19).

### Results

Socio-demographic characteristics of the participants are displayed in Table 1. The majority of participants were males (1387 participants: 73.7%) and single (1253 participants: 66.6%), lived in urban areas (1130 participants: 60%) and had university degrees or postgraduate education (1130 participants: 77.4%). Student participants were 774 (41.1%) and 928 (49.3%) were between 18 and 24 years old during the collection of data period. Participants’ age ranged from 18 to 71 years. Only 10.5% had an acquaintance who was diagnosed with COVID-19.

**Table 1:** Socio-demographic characteristics of participants (*n* = 1882)

Socio-demographic characteristics	Frequency (%)
*Sex*	
Female	494 (26.2%)
Male	1387 (73.7%)
*Age (years)*	
18–24	928 (49.3%)
25–34	460 (24.4%)
35–44	287 (15.2%)
45–54	130 (6.9%)
55–64	59 (3.1%)
65 or more	18 (1%)
*Marital Status*	
Single	1253 (66.6%)
Married	577 (30.7%)
Divorced	42 (2.2%)
Widowed	10 (0.5%)
*Residence*	
Rural	752 (40%)
Urban	1130 (60%)
*Education*	
Elementary or less	87 (4.6%)
Senior High (grades 10–12)	337 (17.9%)
University	823 (43.7%)
Postgraduate	635 (33.7%)
*Occupation*	
Unemployed	90 (4.8%)
University student	774 (41.1%)
Office work (lawyer, sales, education, health)	825 (43.8%)
Construction	193 (10.3%)

*Socio* sociological

Table 2 shows the frequency and percentage of participants who had correct answers for each of the items related to knowledge and attitude regarding COVID-19. It is worth noting that the great majority of participants had good knowledge about the body system affected by COVID-19 (97.4%), while only a 15.4% of participants knew about the incubation period for its causing virus. Around 77% of participants were able to differentiate between COVID-19 and common influenza in terms of symptoms and vaccine effectiveness. Males had better knowledge scores than females in some questions about knowledge in the survey (with significant differences in scores in the questions related to cause of COVID-19 and symptoms caused by COVID-19), while females had significantly higher scores in the questions related to incubation period and about groups at high fatality risk. Significantly higher knowledge scores were also noted among participants with university or postgraduate education in comparison to groups with lower education for the questions related to the organ affected by infection, symptoms, origin, incubation period, groups at high fatality risk and ability of influenza vaccine to protect against COVID-19 (Table 2).

**Table 2:** Number and percentage of participants’ answers for questions related to knowledge and attitude towards COVID-19

***Knowledge***	n (%)	Male	Female	Below university	University or post-grad.
Cause of COVID-19 (virus)	1806 (96%)	1340 (96.6%)	466 (94.3%)*	400 (94.3%)	1406 (92.1%)
Mode of transmission of COVID-19 (virus)	Good knowledge 621 (33%)	463 (33.4%)	158 (32%)	103 (24.3%)	518 (35.5%)
	Fair knowledge 104 (5.5%)	71 (5.1%)	33 (6.7%)	29 (6.8%)	75 (5.1%)
	Poor knowledge 1156 (61.5%)	853 (61.5%)	303 (61.3%)	291 (68.6%)	865 (59.3%)
Body organ/ system affected (respiratory system)	1834 (97.4%)	1357 (97.8%)	137 (27.7%)	401 (94.6%)	1433 (98.3%)**
Symptoms caused by COVID-19	Good knowledge 1018 (54.7%)	775 (55.9%)	243 (49.2%)*	214 (50.5%)	804 (55.1%)**
	Fair knowledge 161 (8.7%)	104 (7.5%)	57 (11.5%)	46 (10.8%)	115 (7.9%)**
	Poor knowledge 682 (36.6%)	494 (35.6%)	188 (38%)*	153 (36.1%)	529 (36.3%)**
COVID-19 first found (animal)	643 (34.2%)	467 (33.7%)	176 (35.6%)	113 (26.6%)	530 (36.3%)*
Incubation period (6–7 days)	290 (15.4%)	182 (13.1%)	108 (29.9%)**	41 (97.6%)	249 (17.1%)**
Possibility of recovery from COVID-19 (yes or in some cases)	1777 (94.4%)	1310 (94.4%)	467 (94.5%)	385 (90.8%)	1392 (91.1%)
Groups at high fatality risk	Good knowledge 1179 (62.7%)	865 (61.7%)	314 (63.6%)*	236 (55.7%)	943 (64.7%)*
	Fair knowledge 95 (5.1%)	56 (4%)*	39 (7.9%)	25 (5.9%)	70 (4.8%)*
	Poor knowledge 607 (32.3%)	466 (33.6%)*	141 (28.5%)	162 (38.2%)	445 (4.8%)*
Similarity in symptoms to influenza virus (Yes)	1458 (77.5%)	1081 (77.9%)	377 (76.3%)	307 (72.4%)	1151 (79%)
Ability of influenza vaccine to protect against COVID-19 (No)	1459 (77.5%)	1077 (77.6%)	382 (77.3%)	276 (65.1%)	1183 (81.1%)**
***Attitudes***					
Perception of danger of infection by COVID-19	Always 349 (18.5%)	230 (16.8%)	119 (24.4%)**	104 (25.1%)	245 (16.9%)**
	Some cases 1494 (79.4%)	1129 (82.2%)	365 (74.8%)	300 (72.5%)	1194 (82.5%)
Concerns to catch COVID-19 (self or family) (yes)	1567 (83.3%)	1161 (83.7%)	406 (82.2%)	339 (80.1%)	1282 (84.2%)*
Readiness to take vaccine (if available) (yes)	1304 (69.3%)	947 (68.3%)	357 (72.3%)	280 (66.2%)	1024 (70.2%)*
Trust in sufficiency of measures taken by government	Yes 993 (52.7%)	728 (53.5%)	265 (54.4%)	208 (50.6%)	785 (54.7%)
	No 523 (27.8%)	387 (28.5%)	136 (27.9%)	126 (30.7%)	397 (27.6%)
Importance of Isolation of COVID-19 cases (Yes)	1827 (97.1%)	1349 (97.8%)	478 (98.4%)	394 (94.7%)	1433 (98.8%)
Optimism about possibility to find a treatment to COVID-19	1700 (90.3%)	1256 (90.6%)	444 (89.9%)	371 (87.7%)	1329 (91.2%)*
Optimism about possibility to find a vaccine to COVID-19	1660 (88.2)	1236 (89.1%)	424 (85.8%)	366 (86.5%)	1294 (88.8%)

*COVID-19* coronavirus disease 2019; * *p*-value < 0.05; ** *p*-value < 0.01; *** *p*-value ≤0.001

As for attitude, the great majority of our participants was found to perceive that infection by COVID-19 is dangerous and that it is important to isolate the cases (97.9 and 97.1%, respectively). The majority were also optimistic about the possibility to find treatment and vaccine to COVID-19 (90.3 and 88.2%, respectively). When it comes to trust in the measures taken by the local government to contain the epidemic, only 19.5% did not have trust, while 27.8% were uncertain and the remaining 52.7% had complete trust in the government implemented measures. Females had significantly higher perception of danger of infection by COVID-19 than males. Furthermore, groups with university or postgraduate education had significantly higher perception of danger, more concern to catch COVID-19 (self or family) and were optimistic about possibility to find a treatment to COVID-19 in comparison with the groups with lower than university education.

Table 3 shows the frequency of practicing preventive measures among participants. The table shows that participants practiced preventive measures to a good extent. The majority of participants answered that they always washed their hands, avoided public places (76.6 and 74.3%, respectively) and disinfected money and food (49 and 66%, respectively). On the other hand, a high percentage (39.2%) answered that they always use personal protective equipment (PPE, such as face/shields and gloves) and disinfect shoes and clothes (49.2 and 46.8%, respectively). Only a small percentage of participants answered that they always went out and always ate raw meat (15.1 and 6.4%, respectively). Additionally, 97.8% of participants knew the ingredients of the disinfectant they use. Males significantly had more frequent positive practices than females in relation to washing hands, avoiding crowds, disinfecting shoes, clothes and lower frequency of going out. Furthermore, participants with university or postgraduate education more frequently avoided crowds and public places with significant differences compared to those with less than university education.

**Table 3:** Percentage of participants as per frequency of preventive practices

***Practices***		Total n (%)	Male n (%)	Female n (%)	Below university n (%)	University or post-grad. n (%)
Washing hands frequently with soap and water	Always	1441 (76.6%)	1083 (78.1%)	358 (72.5%)*	314 (74.2%)	1127 (77.3%)
	Often	197 (10.5%)	132 (9.5%)	65 (13.2%)	53 (12.5%)	144 (9.9%)
	Occasionally	101 (5.4%)	67 (4.8%)	34 (6.9%)	24 (5.7%)	77 (5.3%)
	Never	142 (7.5%)	105 (7.6%)	37 (7.5%)	32 (7.6%)	110 (7.5%)
Avoiding crowds and public places	Always	1398 (74.3%)	1059 (76.4%)	339 (68.6%)**	296 (70%)	1102 (75.6%)**
	Often	215 (11.4%)	141 (10.2%)	74 (15%)	54 (12.8%)	161 (11%)
	Occasionally	95 (5%)	61 (4.4%)	34 (6.9%)	36 (8.5%)	59 (4%)
	Never	173 (9.2%)	126 (9.1%)	47 (9.5%)	37 (8.7%)	136 (9.3%)
Using gloves	Always	737 (39.2%)	569 (41%)	168 (34%)*	175 (41.4%)	562 (38.5%)
	Often	545 (29%)	383 (27.6%)	162 (32.8%)	122 (28.8%)	423 (29%)
	Occasionally	405 (21.5%)	294 (21.2%)	111 (22.5%)	79 (18.7%)	326 (22.4%)
	Never	194 (10.3%)	141 (10.2%)	53 (10.7%)	47 (11.1%)	147 (10.1%)
Disinfecting shoes after going out	Always	926 (49.2%)	726 (52.3%)	200 (40.5%)***	207 (48.9%)	719 (49.3%)
	Often	381 (20.2%)	281 (20.3%)	100 (20.2%)	95 (22.5%)	286 (19.6%)
	Occasionally	297 (15.8%)	193 (13.9%)	104 (21.1%)	63 (14.9%)	234 (16%)
	Never	277 (14.7%)	187 (13.5%)	90 (18.2%)	58 (13.7%)	219 (15%)
Disinfecting clothes after going out	Always	881 (46.8%)	707 (51%)	174 (35.2%)***	218 (51.5%)	663 (45.5%)
	Often	465 (24.7%)	325 (23.4%)	140 (28.3%)	101 (23.9%)	364 (25%)
	Occasionally	329 (17.5%)	220 (15.9%)	109 (22.1%)	57 (13.5%)	272 (18.7%)
	Never	206 (10.9%)	135 (9.7%)	71 (14.4%)	47 (11.1%)	159 (10.9%)
Disinfecting money	Always	923 (49%)	730 (52.6%)	193 (39.1%)***	212 (50.1%)	711 (48.8%)
	Often	410 (21.8%)	287 (20.7%)	123 (24.9%)	87 (20.6%)	323 (22.2%)
	Occasionally	271 (14.4%)	181 (13%)	90 (18.2%)	61 (14.4%)	210 (14.4%)
	Never	277 (14.7%)	189 (13.6%)	88 (17.8%)	63 (14.9%)	214 (14.7%)
Frequency of going out	Always	285 (15.1%)	149 (10.8%)***	136 (27.5%)	47 (11.1%)	238 (16.3%)**
	Often	282 (15%)	174 (12.6%)	108 (21.9%)	53 (12.5%)	229 (15.7%)
	Occasionally	789 (41.9%)	618 (44.6%)	171 (34.6%)	170 (40.2%)	619 (42.5%)
	Never	522 (27.7%)	444 (32.1%)	78 (15.8%)	153 (36.2%)	369 (25.3%)
Disinfecting food	Always	1250 (66.4%)	965 (69.6%)***	285 (57.7%)	266 (62.9%)	984 (67.5%)
	Often	N/A				
	Occasionally	488 (25%)	330 (23.8%)	158 (32%)	115 (27.2%)	373 (25.6%)
	Never	142 (7.5%)	92 (6.6%)	50 (10.1%)	42 (9.9%)	100 (6.9%)
Eating raw meat	Always	121 (6.4%)	70 (5%)***	51 (10.3%)	44 (10.4%)	77 (5.3%)**
	Often	1568 (83.3%)	1190 (85.8%)	378 (%76.5)	329 (77.8%)	1239 (85%)
	Occasionally	191 (10.1%)	127 (9.2%)	64 (13%)	50 (11.8%)	141 (9.7%)
	Never	1568 (83.3%)	0	1 (0.2%)	0	1 (0.1%)

*N/A* not available

* *p*-value < 0.05; ** *p*-value < 0.01; *** *p*-value ≤0.001

Table 4 shows the average knowledge and practices scores per socio-demographic variables and attitudes. Married participants and older age groups had better knowledge scores than the other participants (statistically significant associations with *p*-value≤0.001). Participants in urban areas, those with university or post-graduate education and healthcare workers had higher knowledge scores with *p*-value = 0.009, 0.04 and 0.002, respectively.

**Table 4:** Mean knowledge and practices with socio-demographic variables and attitudes

	Knowledge Mean (S.E.)	*p*-value	Practices Mean (S.E.)	*p*-value
***Gender***				
Male	6.84 (1.35)	0.17	16.69 (4.67)	≤0.001
Female	6.96 (1.34)		17.28 (4.51)	
***Marital status***				
Single	6.75 (1.37)	≤0.001	17.26 (4.73)	≤0.001
Ever married	7.27 (1.27)		16.03 (4.29)	
***Age (years)***				
18–24	6.60 (0.89)	≤0.001	17.32 (3.85)	0.08
25–34	7.09 (1.12)		16.68 (4.11)	
≥ 35	7.22 (1.32)		16.15 (4.28)	
***Live***				
Rural	6.7416 (1.55)	0.009	16.98 (3.76)	0.27
Urban	7.16 (1.02)		16.75 (4.27)	
***Educational***				
Below university	6.78 (0.97)	0.04	17.01 (4.55)	0.01
University or post-grad	7.11 (1.23)		16.43 (3.67)	
***Occupation***				
University student	7.29 (1.12)	0.002	16.11 (3.43)	≤0.001
Others	6.91 (1.54)		16.89 (2.64)	
***Worry about getting infected with COVID-19***				
***Yes***	***6.89*** (0.77)	0.23	***16.79*** (3.28)	0.09
***No***	***6.78*** (0.86)		***17.16 ***(3.76)	

*S.E* standard error, *COVID-19* coronavirus disease 2019

However, although females and those who worry about getting infected with COVID-19 had better knowledge scores, the associations were not statistically significant (*p*-value = 0.17 and 0.23, respectively). On the other hand, males, married participants and healthcare workers had more frequent practices (lower scores) than the other groups (p-value≤0.001). Participants with university or postgraduate education had better practices with statistically significant associations (p-value = 0.01).

## Discussion

Respondents participating in our study answered correctly at an overall average of 67.1% to the questions regarding knowledge of SARS-CoV-2 and COVID-19. However, we found significant deviation from the average depending on the questions asked. Questions about mode of transmission, symptoms, population groups at higher risk and the difference between COVID-19 and the flu were around average (61.5, 54.7, 62.7 and 77.5%, respectively). Questions about the incubation period and the date of the first cases were answered correctly significantly below the overall average (15.4 and 34.2%, respectively). On the other hand, questions regarding the cause of COVID-19, the target body system and its recovery rate were answered correctly at a much higher rate (96, 97.4 and 94.4%, respectively). It might be worth noting that at the time of the collection of data for this study, the predominant opinion about the body system affected in COVID-19 was the respiratory since little information was available implicating other body organs or systems such as blood coagulation [19] and the digestive system [20]. Hence, the appropriate knowledge regarding the afflicted organ was marked based on the widely accepted targeted system at that time. Even if we excluded the answers to the incubation period, our overall average for the knowledge questions will still be at 73%. This finding was correlated to the finding of a similar study conducted in Lebanon during the same period as our study where their overall knowledge average was 75% correct [21]. The correct knowledge averages that we found were comparable to studies done elsewhere. For example, in a Malaysian study, where participants were selected in the same approach compared to ours, the overall correct rate of the knowledge questions was 80.5% [22]. Similarly, correct knowledge was found to be at 90% in China [23] and 81.64% in Saudi Arabia [17]. Our knowledge averages were lower than the aforementioned studied although in our case the data collection was done at a much later time, a time at which global public awareness towards COVID-19 was on the rise. On the other hand, a similar study in Bangladesh found that the correct knowledge average was 54·87% which was less than our findings [24] while another study conducted in Egypt obtained almost the same correct knowledge percentage (71.2%) [18].

Our participants had positive overall attitude towards COVID-19; particularly, the isolation of the COVID-19 cases, optimism about finding a treatment and developing a vaccine (97.1, 90.3, and 88.2%, respectively). This is similar to the attitude of participants in relevant studies [21–23]. However, our sample showed concern about catching the disease (83.3%) and did not seem to have trust in the measures taken by the Lebanese government to manage the COVID-19 epidemic (only 52.7% showed positive attitude towards this question). This latter finding was in sharp contrast with other results where the trust in the country/government of Malaysia and China were 89.9 and 97.1%, respectively [22, 23]. This little trust in government measures regarding the handling of the epidemic is not surprising. Political, economic and social unrest have intensified in Lebanon since October 14, 2019 when people took to the streets demanding a change in the government and the toppling of the ruling parties. Since that date, most of the institutions in the country have become paralyzed leading to immense inflation, and the crippling of the financial as well as the healthcare systems in the country.

At the beginning of the epidemic in Lebanon, the government imposed different measures such as lockdown (from partial to almost complete), closed the borders, created testing and treatment centers as well as announced rules to prevent the spread of COVID-19 related to social distancing and the use of personal protective equipment (PPE). In April 2020, these measures served to limit the spread of the disease, and Lebanon was hailed as one of the successful countries in levelling the graph for new cases [25, 26]. As time passed, especially when the second wave hit the country in the winter of 2020, the government decided to loosen up the measures they imposed at the beginning of the epidemic. They opened the borders and stopped imposing and monitoring the mandatory quarantine for infected incoming travellers. At the same time, they were very loose in enforcing social distancing and the uses of PPE’s in public. During December 2020, restaurants and pubs were allowed to open, albeit with diminished numbers, but in the absence of any enforcement of the rules, social distancing was barely observed. As the government was discussing the need to balance the health benefits of locking down the country versus the harm it does to the economy, numbers of COVID-19 were soaring. The economic difficulties played a major counter role against government-imposed regulation. As an example, the Lebanese government imposed a nationwide lockdown between from November 14 to 29, 2020 [27]. This lockdown was neither respected by the working class nor enforced by the government. As a result, the average of daily confirmed cases in November were 1604 despite the announced lock down [26].

With low numbers of COVID-19 cases in early 2020, the government allocated a single large government run hospital for such cases. As the cases became more spread, more government run hospitals were reserved for treating COVID-19. In addition, the government agreed with private hospitals to allocate part of their facilities for the same purpose. In January 2021, all hospitals have reached critical capacity where they cannot handle new COVID-19 cases [28]. Coupled with more than four thousand average daily new cases, this does not bode well for the control of the disease in the months of February to June 2021.

Our sample participants did not exhibit in high percentages good practices. Only 76.6% washed their hands frequently with soap and water which is less than what others have found in similar studies [22]. Regarding the use of PPE’s, such as gloves, masks or face shields, we found that only 39.2% of our sample always used them. This is a low percentage when compared to the 98.0% of people always wearing facemasks when going out in the Chinese study [23], 95.45% in Bangladesh [24] and even the 51.2% that was found by Azlan et al. 2020 [22]. We cautiously compared our results with studies done countries such as China since the use or PPE’s, such as face masks, has long been a normal practice there. This fact might bias the population towards better adherence to such practice. This low percentage in our study could be attributed to the lack of clear public policy by the Lebanese government which did not impose PPE’s at the time our data was collected (the government implemented the lock down and prevention campaign at the time where this survey was conducted, but the control of the implementation of this strategy was not strict). Similar to what others have found regarding social distancing [22], our sample did observe acceptable social distancing with 74.3% avoiding crowded places and only 15.1% stating that they always went out in spite of the epidemic. At the same time, we found that around half of our sampled participants did disinfect personal items after being out such as their clothes, shoes, and money; while we found 66.4% to disinfect packaged food items they would bring home.

The levels of knowledge and practices we found were significantly higher in people who were ever married, older, with higher education levels, worked in the healthcare sector or related areas and who worry about getting COVID-19. People living in urban areas had significantly better knowledge than those in rural areas without showing a significant difference in practices. Also, gender was not found as a significant factor for knowledge in our studies sample; however, practices were found to be significantly better in males. In most of the studies conducted in this subject, males typically showed less knowledge and attitudes as well as exhibited less cautionary practices compared to females [23, 24, 29]. Although one of these studies was done in a culturally similar environment to the one we have here, we cannot be certain to why we would find that males and females showed insignificantly different knowledge levels with males having better practices. Although this issue needs further investigation, it might be that most of the males in this survey may have been obliged to go to work and not adhere to the lockdown/social distancing since many families in Lebanon rely mainly of males for income. If accurate, this would explain why in this study men were found to have good knowledge compared to females and significantly better practices.

Our findings indicate that the educational background and occupation (the different healthcare fields) correlated positively with knowledge and practices. This finding is not surprising since higher education levels (in medical areas) and healthcare professions are associated with good KAP in most epidemic diseases including COVID-19 [23, 29, 30]. Although the number of healthcare workers surveyed in this study was not very significant, this was to our knowledge the first study to include healthcare workers and nonhealthcare workers in the same questionnaires being asked the same questions and the data analyzed with the same parameters. In spite of the aforementioned shortcoming in numbers, we here cautiously say that healthcare workers exhibited better KAP regarding COVID-19.

Another parameter we looked at regarding knowledge and practices towards COVID-19 was the attitude parameter of how worried our participants were about getting infected. Indeed, we found that being worried about the possibility of being infected with the COVID-19 causing virus does significantly associate with higher knowledge and better practices as has been found elsewhere [31]. On the other hand, it was found that people with health complications and serious concern about getting COVID-19 did not have better knowledge and attitude [32].

### Limitations of the study

This study had few limitations, the main ones being the selection bias related to the surveying method, which excluded all uneducated, illiterate and less technologically savvy individuals. As such, our study included the population sample composed of individuals who were mostly young adults, educated and literate. At the same time, since most participants had a good level of education (95.4% had at least high school degrees or above) and had a second language (English, as a second language) our results may have been skewed towards these two factors. This is unfortunate since the uneducated and elderly might be more susceptible to this disease and therefore should be investigated using a different method.

### Policy implications

Based on the preceding, we suggest further studies into the mechanisms of transmission and dissemination of information to the Lebanese population to try to assess the points of weakness that need to be improved in order to enhance the knowledge of the general populace towards COVID-19 or pandemic level diseases. This should include a detailed analysis of the different media tools and their access by the different socio-demographic tiers of the population.

We would also recommend that governments work towards gaining more trust among the population by putting in place a transparent and a more reassuring public health plan to ensure public safety. It will be interesting to study the comparative KAP of populations in different governments with different economic capabilities to see if having a stronger economy would affect the attitude and eventually the practices of the population in pandemics. Since the world has seen two different examples of how governments supported their people through financial assistance or the lack of it – as happened in Lebanon where the government proposed but barely distributed a very small financial assistance, coupled with unemployment reaching 11.4% many were forced to work in conditions even if they didn’t observe the recommended social distancing [33]– it would, therefore, be recommended for organizations such as WHO to direct more effort towards countries with suffering economies.

Finally, it is our opinion that practices should have been better in Lebanon had the governing bodies in this country been more adamant about enforcing the implementation of rules such as the mandatory wearing of PPE’s and social distancing in public places. We did personally know about cases of social gatherings (weddings, funerals and others) taking place without having any intervention by any of the law enforcement agencies in Lebanon.

## Conclusion

In conclusion, this paper has found overall good KAP levels among the Lebanese people as evident by the control of the COVID-19 spread at the early stages of the disease. On May 5, 2020, the day our data collection ended, Lebanon reported one confirmed case (741 cumulative cases and 25 deaths to that date). Whereas in November 29, 2020, 1696 confirmed cases were reported (125,637 cumulative cases and 991 deaths to that date) [34]. Therefore, we suggest that the lack of trust in the government, due to the lack of economic aid and the absence, or strict enforcement, of the government rules, have led to the increase in the spread of the disease among the people.

## Supplementary Information

**Additional file 1.** KAP-COVID-19 Questionnaire. This file contains the blank questionnaire we distributed among our young population sample based upon which we obtained the data for our current study.

## Data Availability

The datasets generated and/or analyzed during the current study are available from the corresponding author on reasonable request.

## Abbreviations

COVID-19: Corona virus disease 2019
KAP: Knowledge, attitude and practices
MOPH: Ministry of Public Health
SARS-CoV-2: Severe acute respiratory syndrome coronavirus 2
WHO: World Health Organization
