# Supplementary material for: Knowledge, attitude and practices related to COVID-19 among young Lebanese population
Source: BMC Public Health. 2021 Apr 6;21:653. doi: 10.1186/s12889-021-10575-5 (PMC8022301; doi:10.1186/s12889-021-10575-5)
Supplement: Supplementary file 1 — Additional file 1. KAP-COVID-19 Questionnaire. This file contains the blank questionnaire we distributed among our young population sample based upon which we obtained the data for our current study. [file 12889_2021_10575_MOESM1_ESM.pdf]

Knowledge, attitude and practices towards Covid-19 among the Lebanese population.

This survey aims to assess, the Knowledge, attitude, and practices related to the Corona (Covid-19), among the Lebanese community. All the information collected here will stay anonymous and your participation is voluntary. There is no penalty for not participating and no reward for doing so. Also, you can withdraw at any time. You can complete this survey only Once. This survey has been approved by the Institutional Review Board at the Lebanese International University under the reference number LIUIRB-200318-SS1

* Required

Socio-demographic characteristics of Participants

1. 1- What is your gender? *

   Mark only one oval.

   - Male
   - Female

2. 2- What is your marital status? *

   Mark only one oval.

   - Single
   - Married
   - Divorced
   - Widowed

3. 3- What is your age? *

Mark only one oval.

- 18-24 years
- 25-34 years
- 35-44 years
- 45-54 years
- 55-64 years
- 65 years or more

4. 4- Where do you live? *

Mark only one oval.

- In a village (rural)
- In a city (urban)

5. 5- What is your highest educational level? *

Mark only one oval.

- Senior High (grade 10 - 12)
- Bachelor degree
- Masters degree
- PhD degree
- BT
- TS
- other

6. 6- What is your main occupation? *

Mark only one oval.

- Student
- Private business
- Sales man/ woman
- Teacher/ educater
- Driver
- Construction/builder
- Medical/ public health/ health care worker
- Engineer
- Lawyer
- Unemployed
- Other

7. 7- Do you personally know anyone diagnosed positive with Covid-19? *

Mark only one oval.

- Yes
- No

Knowledge assessment

8. 1- What is the cause of Covid-19 (corona)? *

Mark only one oval.

- Virus
- Bacteria
- I do not Know
- Other: ____________________

9. 2- How is Covid-19 transmitted from one person to another? You can choose more than one option. *

Check all that apply.

- [ ] Transmitted through hand shakes and kisses
- [ ] Transmitted by direct contact with patient's personal items
- [ ] Transmitted by sharing the same needle
- [ ] Transmitted by face to face talk
- [ ] Transmitted by blood transfusion
- [ ] Transmitted from a mother to her fetus
- [ ] Transmitted sexually
- [ ] Transmitted by mosquito-bites
- [ ] Transmitted through coughing or sneezing by an infected person
- [ ] Transmitted by consumption of uncooked food
- [ ] Transmitted by touching surfaces contaminated with Corona

10. 3- Covid-19 infects which organ/ system in our body? *

Mark only one oval.

- ( ) Respiratory system
- ( ) Digestive system
- ( ) Eyes
- ( ) Uro-genital system
- ( ) Blood

11. 4- What are the symptoms caused by Covid-19? *You can choose more than one option *

Check all that apply.

- [ ] Abdominal pain
- [ ] Vomiting
- [ ] Skin rash
- [ ] High fever
- [ ] Headache
- [ ] Dry coughing
- [ ] Runny nose
- [ ] Shortness of breath
- [ ] Fatigue
- [ ] Diarrhea
- [ ] Sore throat
- [ ] I do not know

12. 5- The first corona virus causing Covid-19 was found in? *

Mark only one oval.

- ( ) Human
- ( ) Animal
- ( ) Neither in human, nor in animals, it was engineered in the Laboratory
- ( ) I do not know
- ( ) other

13. 6- After being infected, how many days before symptoms of Covid-19 can start to appear in a patient? *

Mark only one oval.

- ( ) 14 days as an average
- ( ) 6-7 days as an average
- ( ) 27 days
- ( ) 40 days
- ( ) I do not know

14. 7- Could an infected person with Covid-19 recover? *

Mark only one oval.

- Yes
- No
- In some cases
- I do not know

15. 8- Who is at high fatality risk (more exposed to death) if caught the Covid-19 ? *You can choose more than one option *

Check all that apply.

- [ ] Children between 1 and 9 years old
- [ ] People above 60 years old
- [ ] Diabetics and cancer patients
- [ ] Immuno-compromised persons (with weak immune system)
- [ ] Pregnant women
- [ ] Persons with heart and lungs problems
- [ ] Everyone

16. 9- What are your sources of information about Covid- 19? *You can choose more than one option *

Check all that apply.

- [ ] World Health Organisation (WHO) website/ recommendations
- [ ] From TV
- [ ] From Newspaper
- [ ] From Family members
- [ ] From Health educators
- [ ] From specialized websites
- [ ] From health care professionals
- [ ] From social media
- [ ] other

Knowledge about viruses

17. 1- Do you think that the influenza virus have the same symptoms as Covid-19? *

Mark only one oval.

- Yes
- No
- Sometimes
- I do not know

18. 2- Do you think that the vaccine for the seasonal influenza (flu) protects against Covid-19? *

Mark only one oval.

- Yes
- No
- Sometimes
- I do not know

Attitude and practices towards Covid-19

19. 1- Do you think that the infection by Covid-19 is dangerous? *

Mark only one oval.

- yes, always
- yes, in some cases
- No
- Do not know

20. 2- Are you worried that you or one of your family members would catch the Covid-19? *

Mark only one oval.

- Yes
- No

21. 3- If a vaccine were available, would you have it? *

Mark only one oval.

- Yes
- No
- Maybe

22. 4- Do you think that the measures taken by the Lebanese government are sufficient to control the Covid-19 pandemic (spread)? *

Mark only one oval.

- Yes
- No
- Maybe
- I do not know

23. 5- What are the measurements that you are taking to prevent being contaminated/ infected by Covid-19? Please indicate all the options (Scale 1-4: 1 for always, 2 for often, 3 occasionally and 4 for never. This for EACH of the below answers) *

Mark only one oval per row.

	1	2	3	4
Washing your hands frequently with soap and water				
Avoiding crowded public places				
Using gloves				
Disinfecting your shoes if you were out				
Disinfecting your clothes if you were out				
Disinfecting money				

24. 6- How often do you go out during this Covid-19 pandemic (spread) ? *

Mark only one oval.

- Always
- Often
- Occasionally
- Never

25. 7- Are you disinfecting food packages you are buying during the Covid-19 pandemic (spread)? *

Mark only one oval.

- Always
- Often
- Occasionally
- Never

26. 8- Are you eating raw meat during the Covid-19 pandemic (spread)? *

Mark only one oval.

- Always
- Often
- Occasionally
- Never

27. 9- Which disinfectant(s) you are using to avoid infection causing Covid-19? *You can choose more than one option *

Check all that apply.

- [ ] Alcohol based
- [ ] Chlorine based (CLOROX)
- [ ] Soap
- [ ] I do not know its chemical property
- [ ] I do not use disinfectants

28. 10- If you have one of the symptoms of the Covid-19 disease? you: *

Mark only one oval.

- Stay at home
- Visit a medical doctor
- Ask for a diagnosis test
- Do nothing and continue normally your life

29. 11- Do you think that the isolation of the suspected cases, with Covid-19, is important? *

Mark only one oval.

- Yes
- No
- Maybe
- I do not Know

30. 12- I am ----------------- regarding the possibility of finding a TREATMENT to Covid-19 *

Mark only one oval.

- optimistic
- pessimistic

31. 13- I am ---------------- regarding the possibility of finding a VACCINE for Covid-19 *

Mark only one oval.

- optimistic
- pessimistic

Thank you

Please note that by clicking on the "Submit" button, you agree to the above-mentioned statements. Your email will be recorded as an e-signature; nevertheless, your identity will be coded and will not be revealed to any third party.

This content is neither created nor endorsed by Google.

Google Forms
